# Medical Residents’ Informal Learning from Pharmacists in the Clinical Workplace

**DOI:** 10.1007/s40670-023-01784-1

**Published:** 2023-05-06

**Authors:** Leslie Carstensen Floren, Amy L. Pittenger, Ingeborg Wilting, David M. Irby, Olle ten Cate

**Affiliations:** 1 School of Pharmacy, University of California San Francisco, 513 Parnassus Avenue, Room S947, San Francisco, CA 94143-0912 USA; 2 School of Pharmacy, University of Minnesota, Minneapolis, MN USA; 3 Utrecht Medical Center Utrecht, Utrecht, Netherlands; 4 Department of Medicine, University of California San Francisco, San Francisco, CA USA

**Keywords:** Resident physicians, Pharmacists, Interprofessional, Clinical workplace learning, Affordances

## Abstract

Workplace-based interactions between residents and pharmacists, though relatively underexplored, might contribute substantially to learning. This international study sought to investigate the affordances residents use for informal learning about medications, their interactions with pharmacists and patterns of resident-pharmacist engagement, as well as residents’ perceived impact of these interactions on their learning. Contextual differences between US and Dutch residency training and electronic health record (EHR) may impact informal learning about medications. We conducted a cross-sectional, online, 25-item survey study, including closed-format and open-response questions among current resident physicians (post-graduate years 1–6, from a variety of residency programs *n* = 803) from the University of California San Francisco, the University of Minnesota, and the University Medical Center Utrecht. Responses from 173 residents in both countries revealed that these physician trainees were afforded opportunities to engage in a wide variety of pharmacotherapy-related activities but engaged differently with social and environmental resources for support. Residents from the United States (US) utilized pharmacists and Up-To-Date, whereas Dutch residents preferentially utilized the online Dutch medication information site and EHR-embedded medication resources. US residents interacted with pharmacists significantly more frequently than Dutch residents. Pharmacists provided residents with a wide range of useful information, much of which is integrated into the medication resources in the Dutch EHR-based decision-support system. While US residents reported overwhelmingly that informal interactions with pharmacists contribute to their learning about medications, Dutch residents’ responses did not confirm this. Intentionally designing residents’ training to include opportunities for interactions with pharmacists could potentially positively impact residents’ informal workplace learning.

**Supplementary Information:**

The online version contains supplementary material available at 10.1007/s40670-023-01784-1.

## Introduction

Most learning in postgraduate medical education happens informally in the clinical workspace. Such learning takes place in the context of everyday work activities, is generally unstructured, often tacit, and may be unplanned or intentional [1–5]. Despite considerable interest in residents’ informal learning processes, gaps exist in our understanding of pharmacists’ contributions to residents’ learning in the clinical environment. Affordances for this interprofessional learning may differ, depending on culture and country. Workplace-based interactions between residents and pharmacists, though relatively underexplored [4], likely contribute substantially to learning. 

Informal learning in the health professions is primarily experiential, occurring as trainees participate in clinical care [6, 7]. According to Billett’s workplace learning theory, learning and working are interdependent and the quality of learning is dependent on: 1) the affordances for learning – including opportunities for learners to participate in relevant workplace tasks and activities as well as access to the support and guidance of experts, co-workers, and resources; and 2) the learner’s engagement with these affordances [8–11]. Informal learning in the clinical workplace is an important contributor to residents’ competency development [4, 12].

Western medicine approaches rely heavily on pharmacotherapy with 66% of US adults and 63% of the Dutch population currently using prescription medications [13, 14]. Resident physicians are expected to develop the knowledge-base and skillset required to plan, implement, and monitor and optimize pharmacotherapy-based treatments (i.e., Accreditation Council for Graduate Medical Education Core Competencies, CanMEDS Physician Competency Framework). Such learning occurs through both formal and informal interactions with pharmacists, other physicians, and interaction with online medical information resources and decision-support tools embedded in electronic health records (EHR). Unfortunately, this learning process is not always sufficient, as evidenced by some practicing physicians’ inadequate drug knowledge and inexperience, which contribute to preventable medication errors, a significant source of morbidity and mortality worldwide [15]. To explore how residents learn pharmacotherapy, we investigated the extent and impact of informal resident-pharmacist interactions in the clinical workplace.

We examined three different health care settings (two in the US and one in the Netherlands) and all years of resident training to answer the following questions: What affordances do residents use for informal learning about medications? How do residents and pharmacists engage with one another in the clinical workplace? And, how do residents perceive the impact of interactions with pharmacists on their learning?

## Methods

We conducted a cross-sectional, online, twenty-one-item survey study among residents at three institutions – two in the US and one in the Netherlands. The Institutional Review Boards of UCSF (#20–31758; Ref. #288894), UMN (#STUDY00010438), and the Netherlands Association of Medical Education Ethics Review Board (NVMO-ERB Dossier #2020.6.4) approved the study.

### Participants and Contexts

We conducted this study among resident physicians of medical and surgical training programs at the University of California San Francisco (UCSF), the University of Minnesota (UMN), and the University Medical Center Utrecht (UMCU) in the Netherlands. US residency training begins after four years of medical training that occurs after four-years of undergraduate study. After secondary school, Dutch medical trainees first complete six undergraduate years, then usually work for multiple years as a ward-physician before starting residency [16]. Relevant for physician’s training regarding medications is that EHR systems in both countries vary substantially. In the US, EHR systems differ depending on the institution and don’t universally contain sophisticated decision-support systems. In the Netherlands, there is a national EHR system that has, embedded within it, online medication resources and a powerful decision-support system (including a drug-drug/drug-patient/drug-gene interaction database; recommended dose adjustments in renal and hepatic compromise; and interpretation of drug levels). This makes the roles of pharmacists different. In the US, pharmacists are often included in ward teams, whereas in the Netherlands, they are available on call but are not generally included in ward teams.

### Survey Development

Following established guidelines [17], we conducted a literature search but found no existing survey related to informal, interprofessional workplace learning. Using Billett’s workplace learning theory, focused on affordances and learner engagement constructs [8–11], we drafted questionnaire items and gathered feedback from three physicians and two pharmacists with training in health professions education research. For expert validation, five panelists – medicine or pharmacy faculty members from the United States, United Kingdom, the Netherlands, and Canada; chosen for their expertise in workplace learning theory, interprofessional education, and pharmacology education – critically reviewed the draft survey to ensure that the constructs of interest were well-represented. Guided by panelist input, we modified the initial draft, producing an online survey including twenty-one closed-response and four open-response questions.

Next, five medical residents (*n* = 2 UCSF, *n* = 1 UMN; *n* = 2 UMCU) completed a cognitive interview while answering survey items [18], with post-interview discussion. We iteratively modified the survey to improve clarity and ensure adequate coverage of workplace learning constructs.

We piloted the survey with three medical residents (*n* = 1, UCSF; *n* = 2 UMN) and one clinical fellow (UMCU) to assess feasibility. Before distribution, we pilot tested the final survey with four residents (*n* = 3 US; *n* = 1, UMCU). The final survey (see Supplementary Material) was distributed via Qualtrics (Qualtrics, 2020).

### Data Collection and Analysis

Data were collected in English during the fall of 2020, in the midst of COVID. The target group of respondents included current medical and surgical residents from UCSF, UMN, and UMCU. A link to the survey, including an explanation of the study purpose and informed consent statement, was distributed by email between October 1 and October 5 to 803 residents at three medical centers (UCSF, *n* = 251; UMN, *n* = 117; and UMCU, *n* = 435), which represent a variety of residents at their institutions (e.g., family and internal medicine, pediatrics, general surgery, OB/GYN programs, and smaller specialty programs). To maximize survey responses [19], residents were sent periodic reminders until survey closure (November 15, 2020). 

Most questions related to residents’ experiences during the prior three months of training, during which time residents may have engaged with pharmacists (either in person or through EHR communications) in multiple clinical rotations. Quantitative questions included either a Likert-type scale or a nominal response format. To minimize the length of the survey while allowing us to gather a broad dataset, some quantitative questions allowed for multiple answers. Questions requesting multiple responses resulted in response counts in excess of the total number of respondents (*n* = 173). For each question, we determined response totals and associated percentages by postgraduate year (PGY) and institution. We performed Chi-square tests, looking for associations between the variable of interest, PGY, and institution. When data from the US institutions were not substantially different, US populations were combined for statistical analysis.

We analyzed open-response questions using directed qualitative content analysis [20]. The first author (LCF) developed a coding system. A second author (i.e., either ALP or IW) checked all response codes. Disputes were reconciled through discussion. Code counts for comparison across groups were performed using R [21] and qualitative findings were summarized in narrative form.

## Results

### Participants

Our study population included current resident physicians, PGY1-6, from UCSF, UMN, and UMCU ((*n* = 803; including medical (e.g., internal medicine, peds, psych, etc.), OB/GYN, and surgical residents at UCSF (*n* = 251), UMN (*n* = 117) and UMCU (*n* = 435)). Between mid-October and mid-November 2020, 175 residents initiated the survey. After removing two respondents (1 UMCU pathology resident deemed unlikely to regularly interact with pharmacists and 1 UMN resident with unspecified specialty), statistical analyses were conducted on data from 173 respondents, yielding an overall response rate of 21.5%. This compares well with other online surveys of academics and health professionals [22, 23] (See Table 1).

**Table 1:** Participant Training Year by Institution (*n* = 173)

	**Respondents** ***N*** **(%)**		
**PGY**	**UCSF**	**UMN**	**UMCU**
**1**	14 (32.6)	26 (43.3)	7 (10)
**2**	17 (39.5)	17 (28.3)	7 (10)
**3**	11 (25.6)	9 (15)	18 (25.7)
**4**	-	6 (10)	15 (21.4)
**5**	1 (2.3)	1 (1.7)	13 (18.6)
**6**	-	1 (1.7)	10 (14.3)
**Total (% of total)**	43 (24.9)	60 (34.7)	70 (40.4)

Participating residency programs included: UCSF: General Surgery and Internal Medicine, UMN: Dermatology, Family Medicine, General Surgery, OB/GYN, and Psychiatry, UMCU: Anesthesiology, Cardiology, Clinical Genetics, Clinical Geriatrics, CT Surgery, Dermatology, ENT, Gastroenterology, General Surgery, Internal Medicine, Medical Microbiology, Nuclear Medicine, Neurosurgery, Neurology, OB/GYN, Ophthalmology, Oral-maxillo-facial surgery, Orthopedics, Pediatrics, Plastics, Psychiatry, Pulmonary and Critical Care, Radiotherapy, Rheumatology, and Urology

*PGY* Post-graduate year, *UCSF* University of California San Francisco, *UMN* University of Minnesota, *UMCU* University Medical Center, Utrecht, the Netherlands

While over 70% of resident respondents from the US were in their first two years of training (UCSF (72.1%), UMN (*n* = 71.6%)), only 20% of UMCU respondents were in their first two years of training, which is attributable to the different structure of residency education in the Netherlands.

During the three months before the survey, resident respondents worked inpatient (85.5%; *n* = 148 of 173), in ambulatory clinics (45.7%; n = 79 of 173), or both settings (32.4%; n = 56 of 173).

### Affordances for Learning

#### Resources for Informal Learning about Medications

Residents were asked to select up to three resources for informal learning about medications from a list of ten options (*n* = 398 responses; *n* = 251 US; *n* = 147 Netherlands). Table 2 includes the top-ranked resources used by country and PGY. US residents learned about medications from Up-to-Date (*n* = 66 of 251, 26.3%) and interactions with pharmacists (*n* = 64, 25.5%), followed by senior residents or fellows (*n* = 41, 16.3%). Dutch residents’ resource use varied by PGY. Across PGYs, they learned about medications most often from EHR-embedded resources (n = 51 of 147, 34.7%); from interactions with attendings (*n* = 35, 23.8%) (i.e., PGY1, 3, and 4 + residents); and from interactions with pharmacists (*n* = 26, 17.7%).

**Table 2:** Top-ranked Resources Residents Used to Answer Medication Questions

**Top three resources ranked**^**a**^							
	**PGY**	**Senior resident or fellow**	**Attending / staff physician**	**Pharmacist or pharmacy resident**	**Lexicomp or Micromedex (US residents)**	**Up-to-Date**	**Online medication resources (Dutch residents)**
**US**	**1**	1^b^		1^b^	3	2	
	**2**		3	2		1	
	**3**		3	2		1	
	**4 + **		2^b^	1	3	2^b^	
**Dutch**	**1**	3^b^	2	3^b^			1
	**2**	2	3^b^	3^b^		3^b^	1
	**3**		2	3			1
	**4 + **		2	3			1

^a^Residents chose their top three resources that they turned to most often for their questions about medications. Additional resource options not included in the table were: another peer resident; Google, and original publications. The three most commonly selected options, by country and by PGY, were assigned the ranks of “1,” “2,” or “3,“ as determined by the percentage of residents selecting the option in each PGY category (Note: percentages were calculated by dividing the number of residents selecting the option in each PGY category by the total number of residents in each PGY category)

^b^Rankings marked by an asterisk were split evenly based on the percentage of residents selecting this option

#### Informal Interactions with Pharmacists

Half of the residents (50.3%; *n* = 87 of 173) indicated that a pharmacist was regularly present as a member of the clinical team in at least one clinical setting, though significantly more often in the US than in the Netherlands (X^2^ = 37.2, *p* =  < 0.0001). Nearly three-quarters of the respondents interacted with inpatient pharmacists at least weekly (*n* = 102 of 139, 73.4%). In ambulatory care, resident-pharmacist interactions were relatively infrequent, with over two-thirds interacting less than once weekly (*n* = 90 of 128, 70.3%).

Responding to the open-response question of “What could have led you to interact more often with a pharmacist during your training so far?”, over two-thirds of residents (*n* = 99 of 141; 70.2%) stated that having both easy and regular access to pharmacists during practice, including dedicated opportunities to work together (e.g., having pharmacists available on rounds, as regular members of their clinical teams, and as participants in multidisciplinary meetings) could have led them to interact more often with pharmacists. As one resident [PGY3, surgery, UMN] responded, “…when you are on an ICU service, you interact with the pharmacists multiple times daily. I think I have a lot of good interaction with pharmacists in the hospital.” As another resident [PGY3, medicine, UCSF] described, “I wish we had a pharmacist on every inpatient team who rounds with us.” To a lesser extent, residents focused on increased opportunities for didactics and other educational sessions with pharmacists. Of note, over half of these responses related to more formal learning sessions came from UMCU residents.

Residents were also asked if pharmacists, when asked for advice, had taken time to explain things to them. Most residents, independent of PGY, agreed or strongly agreed (*n* = 140 of 161, 86.9%) that pharmacists had taken the time to explain things to them.

### Learner Engagement with Pharmacists

#### Frequency of Resident-Pharmacist Interactions

Though UCSF-based residents maintained at least weekly contact across training years, PGY1-2 residents at UMN and PGY1 residents at UMCU engaged in significantly more frequent interactions with inpatient pharmacists than did the more senior residents (X^2^ = 34.0, *p* =  < 0.0001). Dutch residents interacted significantly less often with inpatient pharmacists (i.e., ≤ 1x/week) than US-based residents (X^2^ = 68.6, *p* =  < 0.0001) (See Table 3).

**Table 3:** Residents Interacting with Inpatient Pharmacists ≥ Weekly by Institution and PGY

	**PGY1**	**PGY2**	**PGY3**	**PGY4 + **
**Institutions** Total (*n* = 102 of 139; 73.4%)				
**UCSF** (*n* = 37 of 38; 97.4%)	92.9% (*n* = 13 of 14)	100% (*n* = 15 of 15)	100% (*n* = 8 of 8)	100% (*n* = 1 of 1)
**UMN** (n = 47 of 53; 88.7%)	96.2% (*n* = 25 of 26)	93.3% (*n* = 14 of 15)	71.4% (*n* = 5 of 7)	60% (*n* = 3 of 5)
**UMCU** (*n* = 18 of 48; 37.5%)	60% (*n* = 3 of 5)	25% (*n* = 1 of 4)	35.7% (*n* = 5 of 14)	36% (*n* = 9 of 25)

*PGY* Post-graduate year, *UCSF* University of California San Francisco, *UMN* University of Minnesota, *UMCU* University Medical Center, Utrecht, the Netherlands

**Fig. 1:**
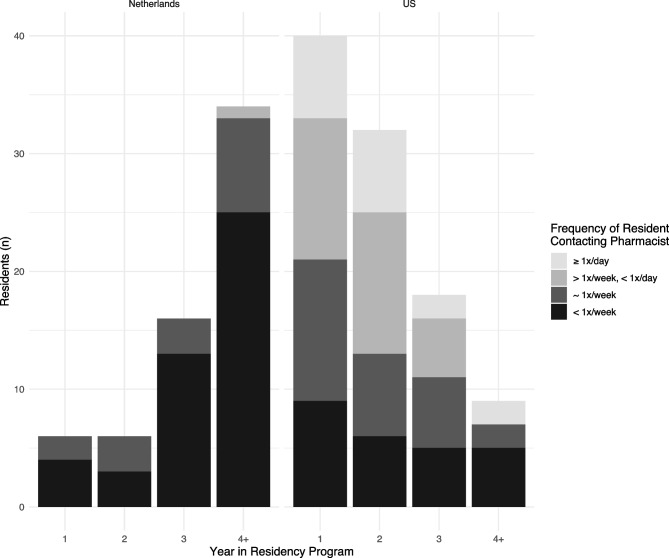
Frequency of Residents Contacting Pharmacists by PGY and Country

US-based residents, especially PGY1s and PGY2s, contacted pharmacists significantly more often than did Dutch residents (*n* = 161 responses; *n* = 62 Netherlands; *n* = 99 US; X^2^ = 46.6, *p* =  < 0.0001). Nearly half of US residents (47.5%; *n* = 47) contacted pharmacists at least multiple times per week, while 98.4% of Dutch respondents (*n* = 61) reported contacting pharmacists ≤ 1x/week. From PGY 3–6, while US residents contacted pharmacists progressively less often, Dutch residents’ contacts increased (X^2^ = 30, *p* = 0.0004) (See Fig. 1).

Pharmacists contacted US residents (*n* = 99) significantly more often than Dutch residents (*n* = 62) (X^2^ = 75.1, *p* < 0.0001), from multiple times per week (*n* = 27, 27.3% vs. *n* = 1, 1.5% Netherlands) up to daily (*n* = 17, 17.2% vs. 0% Netherlands) – especially in PGY1 and 2 (X^2^ = 38, *p* < 0.0001). Dutch residents reported infrequent contacts from pharmacists (i.e., ≤ 1X/week; *n* = 54, 87.1%). Pharmacists commonly contacted residents for questions related to medication or prescription errors and dosing; monitoring drug levels or pharmacokinetic issues; medication availability; drug regimen optimization; and medication selection. Of the twenty-three residents who were never contacted, five were from the US and eighteen were from the Netherlands.

#### Motivation to Engage with Pharmacists

Across PGYs, the most common questions or issues for which US residents contacted a pharmacist were related to dosing (i.e., dose, administration rate, adjustment for organ failure); followed by selecting appropriate medications. Drug side effects and interactions as well as monitoring drug levels and pharmacokinetic issues were also common reasons for all but PGY4 + residents to contact pharmacists. Across training years, Dutch residents’ most common questions were related equally to medication dosing, side effects and interactions; followed by drug-level monitoring and pharmacokinetic issues; and, lastly, drug availability. Eighteen residents (*n* = 13 Netherlands; *n* = 5 US) never contacted a pharmacist.

#### Modes of Engagement for Learning

Residents were asked to select from a list of six common ways that they had learned from pharmacists in the past three months. The most common ways that residents learned from pharmacists (*n* = 293 responses; *n* = 208 US; *n* = 85 Netherlands), varied significantly by country (X^2^ = 25.3,* p* < 0.0001) but were independent of PGY, included asking questions ((*n* = 119 of 293, (*n* = 79 US and *n* = 40 Netherlands), 40.6%)), caring for patients together ((*n* = 63 (*n* = 55 US and *n* = 8 Netherlands), 21.5%)), and engaging in discussions ((*n* = 58 (*n* = 44 US and *n* = 14 Netherlands), 19.8%)).

In an open-response survey question, residents – mostly in PG years 1–4 – provided details about other ways that they had learned from pharmacists over the course of their training. The most common general content area that respondents from all institutions mentioned were drug dosing, administration and optimization. This content area was mentioned twice as often by UCSF respondents relative to those from UMN or UMCU. The most common process by which residents from all institutions learned from pharmacists was through caring for patients together (including multidisciplinary meetings and coordination of care) and disease state management. While UMCU residents were 3-5-fold more likely than UMN or UCSF residents to mention didactics and formal education as a mechanism for learning from pharmacists, residents from UMN and UCSF were approximately three-fold more likely than UMCU residents to mention ward rounding – especially in the ICU – as a process by which they had learned from pharmacists. As one UMN resident [PGY 3, surgery, UMN] explained, “Having a pharmacist on rounds in the ICU is extremely advantageous, and it allows us to get real-life clinical experience with various medications.”

### Perceived Learning Impact

#### Perceived Learning Impact of Pharmacist Interactions

**Fig. 2:**
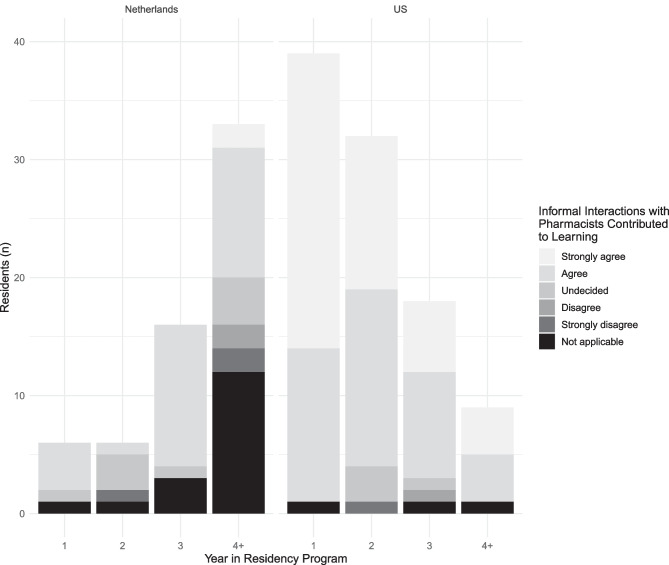
Informal Interactions with Pharmacists Contributing to Learning by PGY and Country

Significantly more US residents (*n* = 89 of 98; 90.1%) strongly agreed or agreed that informal interactions with pharmacists contributed to their learning compared to Dutch residents (*n* = 30 of 61; 49.2%) (X^2^ = 52.0, *p* < 0.0001). US residents’ reported learning from pharmacists was significantly associated with earlier training years (i.e., PGY1-3), independent of specialty (X^2^ = 47.1, *p* < 0.0001) (See Fig. 2).

Pharmacists provided a wide range of information that was useful for residents’ learning needs. PGY1-2 trainees found information related to selection of new medications, dosing, medication errors, and optimization most useful for their learning. Residents in PGY4 + were more likely than PGY1-2 trainees to have found information about side effects, interactions, monitoring drug levels, and pharmacokinetic issues most useful. An open response question revealed a broad range of positive impacts that informal interactions with pharmacists had on residents’ learning including: learning about medications, prescribing, patient care, evidence-based medicine, and drug safety issues.

The majority of resident respondents (*n* = 83 of 99, 83.8% US and *n* = 45 of 62, 72.6% Netherlands) expressed that they would have liked to have had even more opportunities to learn from pharmacists during their training.

### Impact of COVID on Interactions with and Learning from Pharmacists

In two open response questions, we asked residents from all three institutions to report about the impact that COVID-19 had on their interactions with and learning from pharmacists. Approximately one third of responses reflected that the pandemic had had little impact on either their interactions with or their learning from pharmacists during their recent training, but about the same number of responses (i.e., about one third) – mostly from UMN and UCSF medicine residents in PG years 1–3 – reflected that residents’ in-person interactions with pharmacists had been reduced in frequency with a corresponding increase in telephonic communication.

This move away from face-to-face interactions and towards increased usage of phones, pagers, video conferencing negatively impacted learning for a small number of residents. One resident [PGY1, medicine, UMN] said, “When they (pharmacists) aren't in person, it’s so much worse. Less communication. Harder to get a hold of… If they are in clinic less, then I can't ask them my "on the fly" questions where most of my learning is done.”

## Discussion

Residents in both the US and the Netherlands were afforded opportunities to engage in a wide variety of activities and resources related to pharmacotherapy but interacted in a different manner with pharmacists, other physicians, and online information resources for guidance and in support of those activities. US residents relied mostly on pharmacists and Up-To-Date for medication-related questions. Dutch residents relied mostly on the EHR-embedded medication resources. Compared to their Dutch counterparts, US residents primarily interacted with, and learned from, pharmacists during informal, face-to face encounters on the wards. In the Netherlands, residents interact with pharmacists more during on call consultations, multidisciplinary patient consultations, and formal educational settings, especially in the early years. Pharmacists provided a wide range of information useful for resident learning, much of which is incorporated within the Dutch EHR. While US residents report that informal interactions with pharmacists contribute to their learning about medications, Dutch residents’ responses did not confirm this affordance.

To help answer medication-related questions, US-based residents utilized pharmacists as medication information resources more often than Dutch residents. This difference in utilization of pharmacists is likely multi-factorial and impacted by greater accessibility of pharmacists in the US relative to the Netherlands as well as Dutch residents’ substantial pre-training practice experience relative to their counterparts in the US. Dutch residents tend to have more clinical experience than US residents for the same level of postgraduate training because many of them have several years of clinical working experience before entering formal residency training. In addition, most Dutch residents start their early training years in a general hospital setting whereas senior postgraduate years are usually spent at a university hospital setting. The need for interaction might, in addition, also be impacted by the integration of key medication information and decision-support in the Dutch medication resource and EHR.

Residents in both countries relied heavily on indirect support provided by online medication information resources. This finding reflects the growing imperative for evidence-based clinical decision making [24] and highlights the Dutch residents’ ease of access to the Dutch medication information site and EHR-embedded medication resources as an especially important affordance. Our findings parallel those of Chong and colleagues who found that most Australian junior doctors utilized online clinical resources heavily in their daily practice [25]. To support residents’ learning, pharmacists might purposefully model how they integrate online resources in their own clinical decision-making processes.

One of the most salient, and to some extent surprising, findings was the difference in reported resident-pharmacist interaction frequency internationally. While most US residents interacted with pharmacists daily or weekly, Dutch residents interacted much less often with pharmacists. In the US, pharmacists were frequently present on the wards and participated in ward rounds, and informal interactions happened naturally. Rounding with pharmacists was an especially impactful affordance for learning for US-based residents in our study, but rarely occurred in the Netherlands. At UMCU, like many teaching hospitals in the Netherlands, pharmacists are available on-call, attend multidisciplinary patient consultations, and provide therapeutic recommendations to physicians on demand (through phone calls and via chart notes in the EHR) and during patient consultations, but they do not regularly serve on clinical wards. Though Dutch residents highlighted formal didactics from pharmacists as important learning activities, informal interaction with pharmacists was less often recognized as an affordance. Currently, Dutch pharmacists’ regular service on clinical wards is becoming more common in practice, but this is hospital-dependent and certainly not common practice throughout all hospitals.

Will the presence of pharmacists on clinical wards affect the eventual knowledge, skills, and attitudes of residents? While we presume that it might, there are two contrasting hypotheses. One is that, as US residents seem to confirm, frequent interactions with pharmacists in the clinical workplace enhances resident knowledge. The other hypothesis is that Dutch residents, with infrequent opportunities to interact with clinical pharmacists, are forced to engage in active learning by answering their own questions. While we did not test these hypotheses, we recognize that integrating Dutch pharmacists in daily ward rounds would promote interactions between pharmacists and residents. Whether such increased interprofessional interactions on rounds would be viewed by Dutch residents as a learning affordance (as did the US residents) and/or would improve Dutch residents’ learning from pharmacists remains to be studied [26, 27].

A majority of residents across contexts reported that pharmacists took time to explain things to them and they wanted even more opportunities to learn from pharmacists. This comports with Noble and colleagues’ finding that direct instruction and guidance, assistance in decision-making, and feedback from pharmacists were key affordances supporting residents’ learning to prescribe [28]. Similarly, Tubb & Loesch reported that internal medicine residents who interacted with pharmacists in acute care rounds “always” wanted a pharmacist team member, appreciated pharmacists’ recommendations, and felt that these interactions had improved their drug knowledge [27]. However, a small contingent of residents, mainly from the Netherlands, never contacted a pharmacist. Reasons for lack of engagement with pharmacists may include: lack of opportunity to interact; lack of awareness that pharmacists could be useful learning resources; greater clinical experience of entry-level Dutch residents compared to entry-level US residents; more readily available clinical decision-making tools and information provided through the Dutch EHR; or a workplace microculture and organization of health care (i.e., fewer number of Dutch pharmacists in clinical care and greater involvement of pharmacy technicians with a broader scope of practice than pharmacy technicians in the US) that does not support resident-pharmacist interaction [29, 30]. For some residents, especially in the US, COVID safety protocols limited their interactions with pharmacists. Residents in the current study suggested brief internships, ward rounds, and presence in clinic as mechanisms to increase opportunities for interactions with pharmacists.

While most studies related to pharmacist-physician interactions have focused on junior doctors’ prescribing practices [28, 31–33], we characterize how residents across training years engage with pharmacists, formularies and EHRs during pharmacotherapy-related activities and how they value these interactions for learning. We have also provided an international context by exploring the impact of practice differences across countries and how these differences reflect available affordances. Our study has identified that pharmacists are an affordance for residents’ learning in the clinical workplace that, in some cases, is not fully utilized and highlights the need to train current pharmacists as well as pharmacy learners to assume this important role as resident educators. Since we found that pharmacists serve as important learning resources for some residents, future research may focus on identifying interventions to enhance access to and utilization of pharmacists to optimally support residents’ learning and on identifying how informal physician- pharmacist interaction influences knowledge, skills, and attitudes towards pharmacotherapy and, thereby, influences professional performance.

The primary limitations of our study were the limited access to some resident populations for recruitment at the US institutions as well as the low response rate at UCSF and UMCU. Although the number of respondents was relatively equal across institutions, the disproportionate number of surveys sent out between the institutions was unequal and could have skewed the data. The low response rate also creates the potential for bias (i.e., non-responder and responder bias) and limits the generalizability of our findings. However, we believe that the patterns of behavior reported by residents and our interpretations of them offer useful insights into workplace learning between pharmacists and residents. The reasons for the low response rates are most likely multifactorial and may include resident prioritization of clinical duties during the fall of 2020 with COVID-19 surges as well as survey fatigue [34]. Since we did not measure residents’ pharmacotherapeutic knowledge and skills, we are unable to draw conclusions about the direct effects of resident-pharmacist interactions on resident learning.

## Conclusion

Informal, interprofessional workplace learning occurs in the midst of residents’ interactions with pharmacists. For some medical residents, pharmacists have been identified as significant contributors to their development of pharmacotherapeutic knowledge. Dutch residents’ informal interactions with pharmacists were more limited and the Dutch medication information site and electronic health record-embedded medication resources, as well as formal education, served instead as important affordances for their learning about medications. Intentionally designing residents’ training to include opportunities for interactions with pharmacists could potentially impact residents’ informal workplace learning, especially in the US. To what extent Dutch residents’ learning would benefit from these interactions remains to be further studied.

## Supplementary Information

Below is the link to the electronic supplementary material.

Supplementary file1 (DOCX 23 KB)
