# Supplementary material for: Medical Residents’ Informal Learning from Pharmacists in the Clinical Workplace
Source: Med Sci Educ. 2023 May 6;33(3):701–10. doi: 10.1007/s40670-023-01784-1 (PMC10163287; doi:10.1007/s40670-023-01784-1)
Supplement: Supplementary file 1 — Supplementary file1 (DOCX 23 KB) [file 40670_2023_1784_MOESM1_ESM.docx]

**SUPPLEMENTARY MATERIALS**

*Physician-Pharmacist Interaction Survey*

**Introductory Information**

*Principal Investigator:* XXX

We invite you to participate in a voluntary **research study** to **explore residents’ learning from** **interprofessional interactions with pharmacists in the clinical workplace.** 

Our goal is to survey residents at all levels of postgraduate training during the 2020-2021 academic year from three universities (i.e., US University 1, US University 2, and the Dutch University) about their experiences in interacting with pharmacists in the clinical workplace.

Through this survey study, we hope to gain knowledge about residents’ interactions with pharmacists and to better understand how these interactions may impact residents’ informal learning.

The investigators have no financial or proprietary interests.

**What will happen if I take part in this study?**
If you agree to participate in this study, you will complete an online survey with questions about your experiences interacting with pharmacists during your training. Some questions will focus on the last 3 months of training, while others will ask you to think back over the full course of your residency training so far. The survey will take about 10-15 *minutes* to complete.

**Can I stop being in the study?**
Yes. Participation in this study is optional. You can skip questions that you do not want to answer or stop the survey at any time.

Your participation will not affect your educational or future status at US University 1, US University 2 or the Dutch University.

**Will information about me be kept private?**
We will keep your answers confidential and will not share your personal information with anyone outside the research team. The team consists, next to the principal investigator from US University 1, Dr. YYY (US University 2), Drs. ZZZ and AAA (both the Dutch University).

**Who can answer my questions about the study?**
Please contact the Principal Investigator, Dr. XXX at (email address) . If you have questions or concerns about your rights as a research participant, you can call the US UNIVERSITY 1 Institutional Review Board at (phone number). The study has been approved by the US UNIVERSITY 1 Institutional Review Board (#XXX), the Netherlands Association of Medical Education Ethics Review Board (NERB Dossier #XXX) and the US University #2 Institutional Review Board (#XXX).

**Informed Consent Questions:**

Please note that [If “No” is selected, the following statement will display: Please contact the Principal Investigator, Dr. XXX at (email address) before continuing.]

I have read the information letter and I have had the opportunity to ask questions about this study, either in writing (i.e., by email) or by phone. My questions have been sufficiently answered and I have had sufficient time to consider participating.

- Yes
- No

I acknowledge that my participation is voluntary and that I can retract at any time without consequences. I am aware that I am not obliged to give a reason for retracting myself from participating in the study.

- Yes
- No

 I am aware that participating the study is voluntarily and that I can stop participating at any time during the study.

- Yes
- No

|  |
| --- |

I know that in case I retract from participating in the study, any data that I have provided thus far can be used for analysis unless I specifically ask to destroy any data that I have provided up till the moment I retract from participating. Destruction of my data is only feasible in case this does not harm the study.

- Yes
- No

I give permission to collect, store and use my answers to allow answering the research question of this particular research study. 

- Yes
- No

I am aware that, in order to allow for checking scientific integrity, some people may be granted permission to access the gathered data.

- Yes
- No

I am willing to participate in this study.

- Yes
- No

**Begin Survey**

| **Note 1:** In some questions, the terminology of 'informal learning' is used. Informal learning includes interactions with pharmacists and pharmacy residents that may occur in the course of day-to-day clinical service but are not explicitly planned for educational purposes.  **Note 2:** When the word 'pharmacist' is used, it includes pharmacy residents but *not* pharmacy technicians or assistants.  **Note 3**: We realize that the past 3 months may not be typical for your clinical work. For those questions focusing on the last three months of your residency, we would like you to answer the questions just as reflecting the past 3 months, and *not* how things or interactions *should* have happened. In last two questions, we will ask you how the COVID-19 pandemic has affected your interactions with pharmacists. |
| --- |

1. Select your academic affiliation:

- US University 1
- US University 2
- Dutch University

2. Select your year in the program:

- 1
- 2
- 3
- 4
- 5
- 6
- 7

3. What is your residency specialty?

- Anesthesiology
- Cardiology
- Cardiothoracic surgery
- Critical care and intensive care medicine
- Dermatology
- Diagnostic radiology
- Emergency medicine
- Family medicine
- Gastroenterology
- General surgery
- Genetics or clinical genetics
- Geriatrics
- Hospital medicine
- Internal medicine and subspecialties
- Laboratory medicine
- Oral and maxillofacial surgery
- Microbiology
- Neurology
- Neurosurgery
- Nuclear medicine
- Obstetrics/Gynecology
- Ophthalmology
- Orthopedic surgery
- Otorhinolaryngology
- Pathology
- Pediatrics
- Psychiatry
- Plastic surgery
- Pulmonology / respiratory medicine
- Radiotherapy
- Rehabilitation medicine
- Rheumatology
- Sports medicine
- Urology
- Other

5. In at least one clinical context where you have worked in the past 3 months, is there a pharmacist who is included as a regular member of the clinical team?

- Yes
- No

6. To which resource do you turn *most often* if you have questions about medication?
[Check up to 3]

- Another peer resident
- A senior resident or fellow
- An attending / staff physician
- A nurse
- A pharmacist or pharmacy resident
- Google search (e.g., Wikipedia or other sources)
- Lexicomp or Micromedex (US residents)
- Online specialty formulary (Dutch residents – including farmacotherapeutisch compas)
- Original publication (e.g. via PubMed)
- Up-to-Date

7. Which modes of interaction with a pharmacist have you used most frequently in the past 3 months?
*[Check up to 3]*

- Electronic Health Record
- In person (face to face)
- Phone call
- SMS / text message / what's app
- Email
- Web-based messaging / Voalte / My Chart
- Video conferencing

8. How often did *you* contact a pharmacist in the past 3 months?

- Less than once per week
- About once per week
- Less than once per day but multiple times per week
- About once per day
- Multiple times per day

9. For which type of questions or issues did *you* contact a pharmacist most often in the past 3 months?
*[Please read all possible answers, then check up to 3]*

- I did not contact a pharmacist
- Selecting appropriate new medication
- Medication or prescription errors
- Medication availability
- Medication dosing (dose, rate of administration, adjustment for organ failure)
- Drug administration routes (oral, IV, subcutaneous, etc.)
- Optimization of existing drug regimen
- Insurance issues
- Drug side effects and interactions
- Monitoring drug levels/ pharmacokinetics
- Patient education and information
- Drug intoxication, drug overdose or poisoning
- Transition of care to other setting
- Preparation of drug formulation / drug compounding
- Other

10. How often did *a pharmacist* contact you in the past 3 months ?

- Less than once per week
- About once per week
- Less than once per day but multiple times per week
- About once per day
- Multiple times per day

11. For which type of questions or issu*es* did *a pharmacist* contact you most often in the past 3 months?
*[Please read all possible answers, then check up to 3]*

- I was not contacted by a pharmacist in the past 3 months
- Selecting appropriate new medication
- Medication or prescription errors
- Medication availability
- Medication dosing (dose, rate of administration, adjustment for organ failure)
- Drug administration routes (oral, IV, subcutaneous, etc.)
- Optimization of existing drug regimen
- Insurance issues
- Drug side effects and interactions
- Monitoring drug levels/ pharmacokinetics
- Patient education and information
- Drug intoxication, drug overdose or poisoning
- Transition of care to other setting
- Preparation of drug formulation / drug compounding
- Other

12. Which information have you received from a pharmacist that was *most useful* for your learning needs in the last 3 months?
*[Please read all possible answers, then check up to 5]*

- I did not receive information from a pharmacist in the past 3 months
- Selecting appropriate new medication
- Medication or prescription errors
- Medication availability
- Medication dosing (dose, rate of administration, adjustment for organ failure)
- Drug administration routes (oral, IV, subcutaneous, etc.)
- Optimization of existing drug regimen
- Insurance issues
- Drug side effects and interactions
- Monitoring drug levels/ pharmacokinetics
- Patient education and information
- Drug intoxication, drug overdose or poisoning
- Transition of care to other setting
- Preparation of drug formulation / drug compounding
- Other

13. Informal interactions with pharmacists have contributed to my learning about medications in the past 3 months.

- Not applicable
- Strongly disagree
- Disagree
- Undecided
- Agree
- Strongly agree

14. When asked for their advice, most pharmacists I have worked with have taken time to explain things to me in the past 3 months.

- Not applicable
- Strongly disagree
- Disagree
- Undecided
- Agree
- Strongly agree

15. Please check the most common ways you have learned from pharmacists over the past 3 months.

[In next question, you will have an opportunity to specify any other ways you have learned from interacting with pharmacists during your residency training *so far.*]

- I have not learned from interacting with pharmacists over the past 3 months
- Observing them as they work
- Asking them direct questions
- Caring for patients together
- Engaging in discussions with them (i.e., about patient issues, drug trials, etc.)
- Receiving feedback from them

16. Please state in which other ways you have learned from pharmacists during *all of your residency training so far*, or please leave blank if not applicable.

17. Would you have preferred to have more opportunities to learn from pharmacists during your training so far?

- Yes
- No

18. Please tell us about what could have led you to interact more often with a pharmacist during *all of your residency training so far.*

19. Please tell us how interactions with pharmacists have impacted your learning during *all of your residency training so far.*

Over one half year the COVID-19 pandemic has severely affected health care, training and inter-professional interactions.

 20. Please tell us how this has affected your interactions with pharmacists.

 21. Please tell us how this has affected your learning from pharmacists.
